# A rapid and reproducible method for generating germ-free *Drosophila melanogaster*

**DOI:** 10.52601/bpr.2024.240025

**Published:** 2024-12-31

**Authors:** An-Qi Li, Sha-Sha Li, Hui-Lin Li, Lan-Lan Zhong, Guo-Bao Tian, Xin-Yuan Zhao, Qiao-Ping Wang

**Affiliations:** 1 Department of Pharmacy, The Third Affiliated Hospital, Sun Yat-Sen University, Guangzhou 510630, China; 2 Lab of Metabolism and Aging, School of Pharmaceutical Sciences (Shenzhen), Shenzhen campus of Sun Yat-sen University, Shenzhen 518107, Guangdong, China; 3 Department of Immunology, School of Medicine, Sun Yat-Sen University, Shenzhen 518107, Guangdong, China; 4 Medical Center for Comprehensive Weight Control, The Third Affiliated Hospital of Sun Yat-sen University, Guangzhou 510630, China; 5 Guangdong Provincial Key Laboratory of Diabetology, Guangzhou Key Laboratory of Mechanistic and Translational Obesity Research, The Third Affiliated Hospital of Sun Yat-sen University, Guangzhou 510630, China

**Keywords:** Axenic, Germ-free, Microbiota, *Drosophila*, Protocol

## Abstract

Microbial communities exert a profound influence on various facets of animal behavior and physiology, making the comprehension of their interactions with hosts or the environment essential. *Drosophila melanogaster*, a widely recognized model organism, has been pivotal in elucidating host-microbe interactions. Despite the existence of several protocols for generating germ-free (GF) *Drosophila*, their reproducibility has been constrained by the technical difficulty of maintaining airtight conditions in centrifuge tubes. In this study, we introduce a refined method for the production of GF *Drosophila*, complemented by a straightforward verification process to ascertain its efficacy. We propose an innovative strategy employing bio-reaction tubes equipped with a 0.22 μm filter membrane cap, which facilitates the rearing and maintenance of GF flies, thereby streamlining the procedure and enhancing the efficiency of model construction.

## INTRODUCTION

The significant influence of microbial communities on host physiology (Ogunrinola *et al.*^ 2020^) and behavior (Kim and Shim 2023; Vuong *et al.* 2017) has become increasingly recognized in recent years. Understanding these host-microbe interactions is therefore paramount. Germ-free (GF) animal models serve as valuable tools to elucidate the role of symbiotic microbiota in organismal development and function, providing insights into host-microbe interactions in both health and disease states.

One of the strengths of *Drosophila* as an important model for studying the effects of microbiota on host health is the ability to produce large quantities of GF flies and flies with specific gut microbiota in a straightforward, standardized way, which can significantly increase the efficiency of experiments. Several studies have successfully established standardized procedures for GF fly production (Bronnec and Alexeyev 2022; Kietz *et al.* 2018; Koyle *et al.* 2016) which typically involve briefly incubating flies’ eggs in hypochlorite solution to eliminate the microbiota. The sterile eggs are then transferred to centrifuge tubes containing sterile food to prevent microbial contamination.

However, conventional methods utilizing sterile centrifuge tubes with sterile food often necessitate frequent loosening of the caps to maintain air exchange. This human intervention introduces inconsistency and increases the risk of microbial contamination, compromising both the productive efficiency and model stability of GF flies. This protocol proposes a simpler and more stable approach for GF fly preparation by introducing bio-reaction tubes equipped with 0.22 μm filter membrane caps. These tubes facilitate air exchange while effectively preventing microbial entry, creating a more optimal environment for GF fly rearing.

## MATERIALS

The materials used in this method are listed in Table 1.

**Table 1:** The materials used in this method

Material	Source	Identifier
Agar	Biofroxx	8211GR500
Corn flour	Shandong Dongying grain and oil store	/
Yeast	Angel	BQT00380
Brown sugar	Yaoma ecological farm	/
Propionic acid	Sigma	P5561-1L
Methylparaben (10%)	Sigma	H5501-100G
High-temperature tissue culture sealing film	Biosharp	BS-QM-01B
Bio-reaction tubes	Biofil	BRT010050
Grape juice	Huiyuan Juice	/
Brush	Difannier	Line drawing pen
100 μm cell sieves	Biosharp	BS-100-XBS-N1
Sodium hypochlorite solution (3.3%)	Damao	/
MRS	HKM	027312
Ethanol (75%)	Guangzhou Chemical Reagent Factory	64-17-5
Breathable mesh	Tangzheng Home	80 mesh Nylon net yarn
Electric homogenizer	Sangon	G50600-0001

## PROTOCOL

### Sterile food preparation

1 Weigh 3 g of agar, 18.75 g of corn flour, 20.625 g of brown sugar, and 7.35 g of yeast into a 500 mL conical flask.

2 Add water to the conical flask to reach a total volume of 300 mL, and mix the contents thoroughly.

3 Boil the mixture in a microwave oven for 5 min, ensuring it reaches a boil three consecutive times.

4 Cover the mouth of the conical flask with high-temperature tissue culture sealing film, followed by tinfoil.

5 Sterilize the mixture using an autoclave at 121 °C for 20 min.

**Figure 1:**
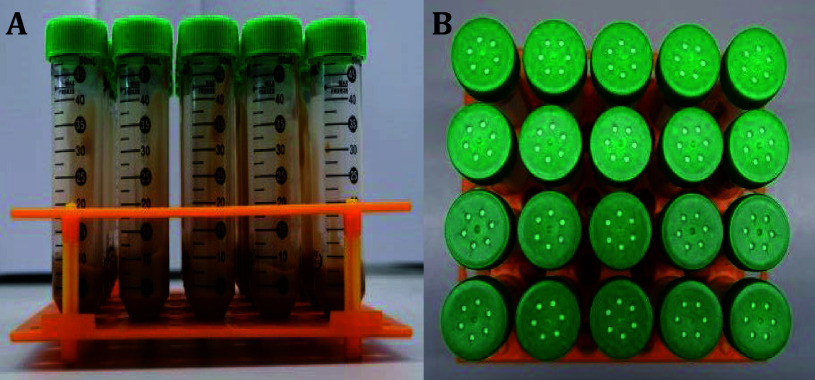
Sterile food in bio-reaction tubes. **A** Front view. **B** Top view

6 Add 1.8 mL of propionic acid and 3.6 mL of 10% methylparaben to the sterile diet on an ultra-clean bench. Mix well, and transfer 7.5 mL of the diet into 50 mL bio-reaction tubes with 0.22 μm filter membrane caps (Fig. 1).

7 Allow the diet to cool on the ultra-clean bench overnight and store at 4 °C for use within one week.

### Egg collection

#### Preparation of grape juice medium plates

1 Weigh 25 g of agar dissolved in 500 mL of double-distilled water and boil the mixture in a microwave.

2 Add 300 mL of grape juice to boiled agar and continue to boil together for 15 min, then cool to 60 °C.

3 Add 18 mL of 10% methylparaben, mix well, pour 20 mL into 100 mm petri dishes, let cool until completely solidified, and store at 4 °C.

#### Assembly of egg collection cages

1 Cut off the bottom of a clear plastic bushing and make a square cut in the center of the lid, with each side measuring 3 cm.

2 Cut a breathable mesh slightly larger than the incision, and fix the filter mesh above the incision with tape.

3 Mix 1 g of yeast with 15 g of water to form a yeast paste and apply it to the center of the grape juice medium plates.

**Figure 2:**
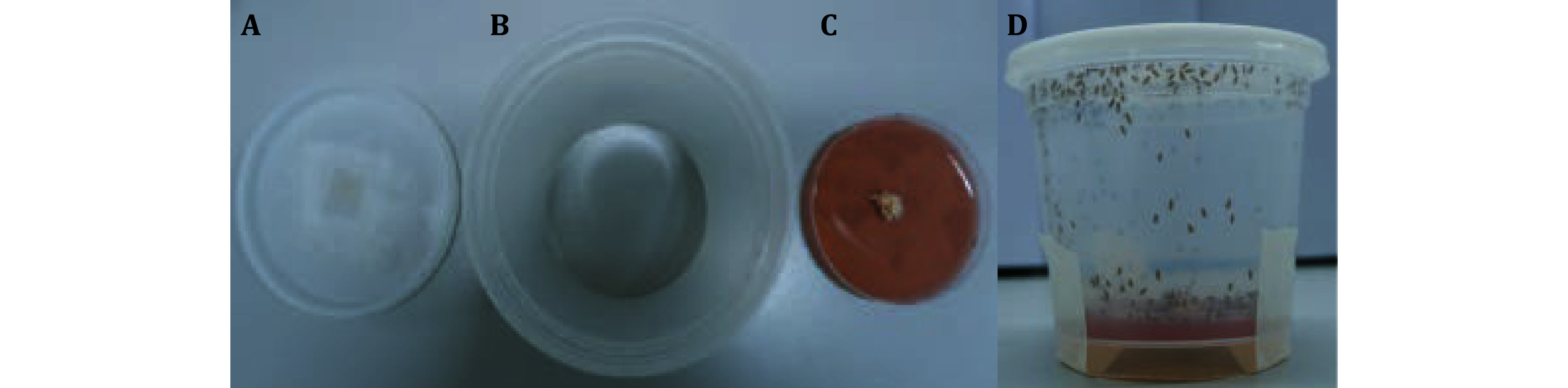
Assembly of egg collection cages. **A** The lid of egg collection cages. **B** The side walls of the egg collection cages are formed by cutting off the bottom of a clear plastic bushing. **C** The grape juice medium plates with yeast paste. **D** The fully assembled egg collection cage

4 Place the grape juice medium plates with yeast paste into the plastic bushing cut off at the bottom to form an egg collection cage, securing the bottom with tape (Fig. 2).

#### Setup of parental flies

1 Anesthetize 4–7 days wild-type flies using carbon dioxide.

2 Transfer 150 males and females to egg collection cages and incubate at 25 °C for 18 h.

3 Discard the grape juice medium plate with collected eggs, transfer the flies to a new egg collection cage, and incubate at 25 °C for another 18 h.

#### Egg collection

1 Remove all parental flies from the egg collection cages and extract the plate of grape juice medium plate with eggs.

2 Under a microscope, remove hatched larvae and parental fly carcasses from the grape juice medium plates, along with the yeast paste.

### Dechorionation of eggs and transfer to the sterile diet

**Figure 3:**
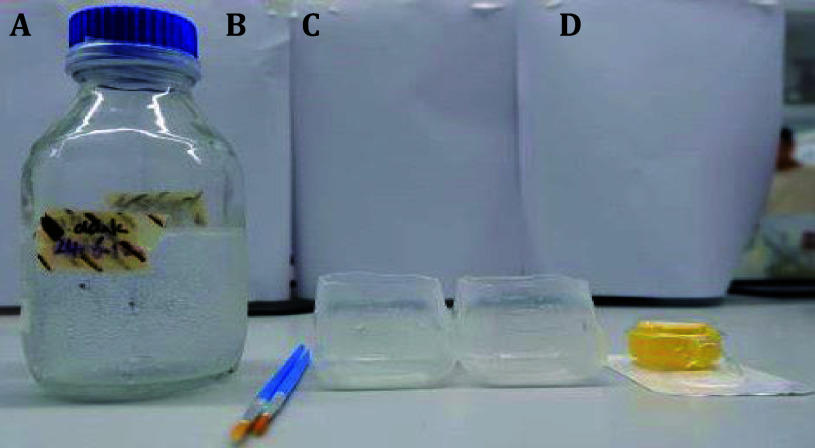
Washing kit for egg dechorionation. **A** Sterile double-distilled water. **B** Sterile brushes. **C** Egg-washing containers. **D** 100 μm cell sieves

1 Place a sterile brush, sterile double-distilled water, 100 μm cell sieves, and egg-washing containers under UV light for 15 min (Fig. 3).

2 Transfer the grape juice medium plate containing eggs to a clean bench. Add sterile double-distilled water to the plate to wash the eggs. Gently brush the eggs on the Petri dish surface with a sterilized brush, pour the slurry on a 100 μm cell sieve, and repeat this process 3–4 times until all eggs are transferred to the cell sieve.

3 Pour 25 mL of 3.3% sodium hypochlorite solution into a sterile container. Pick up the cell sieve containing the eggs with forceps and move it up and down in the sterile container to wash the eggs for 2.5 min.

4 Transfer the cell sieve with the eggs to a second sterile container pre-filled with 3.3% sodium hypochlorite solution and repeat the washing process for another 2.5 min.

5 Move the cell sieve with eggs to a sterile container pre-filled with sterile double-distilled water and wash eggs for 2.5 min.

6 Transfer the cell sieve with eggs to a second sterile container pre-filled with sterile double-distilled water and repeat the washing process for another 2.5 min.

7 Use a sterile brush to pick up 30–50 eggs and transfer them to sterile food in bio-reaction tubes with 0.22 μm filter membrane caps, then place the bioreaction tubes in an incubator at 25 °C.

8 Five days after transferring the sterile eggs to the bio-reaction tubes, insert sterile paper towels into the sterile food to absorb moisture and larvae crawling.

### Germ-free fly generation and verification

#### Preparation of solid MRS medium

1 Weigh 5.4 g of agar and 16.2 g of MRS powder in 300 mL double-distilled water, and mix thoroughly.

2 Sterilize the medium by autoclaving at 121 °C for 20 min. Pour 20 mL into each 100 mm petri dish on a clean bench, and cool for 30 min until completely solidified.

3 Seal the Petri dish with sealing film and store them at 4 °C.

#### Preparation of liquid MRS medium

1 Weigh 16.2 g of MRS powder in 300 mL double-distilled water and mix them well.

2 Sterilize the medium by autoclaving at 121 °C for 20 min. Cool the medium to room temperature, and store it sealed at 4 °C.

#### Extraction of adult flies and washing with ethanol

1 Ten days after transferring sterile eggs to bio-reaction tubes observe larvae emerging as adults. Anesthetize the adults by freezing them.

2 Transfer 3–5 adult flies to 1.5 mL sterile centrifuge tubes containing 200 μL 75% ethanol solution. Pipette up and down to wash the flies for 30 s, then discard the ethanol.

3 Wash the fruit flies twice with 200 μL clean, sterilized 75% ethanol, and discard the liquid.

#### Washing with Sterile water

Add 200 μL of sterile double-distilled water to the centrifuge tube containing the flies. Pipette up and down to wash the flies for 30 s, then discard the water.

#### Washing with sterile liquid MRS medium

Add 200 μL of sterile liquid MRS medium to the centrifuge tube containing the flies. Wash the flies for 30 s, then discard the medium. Repeat this step twice.

#### Homogenization of Flies

Add 200 μL of sterile liquid MRS medium to the centrifuge tube containing the flies. Homogenize the flies using an electric homogenizer until thoroughly ground.

#### Inoculation on solid MRS medium and observation of results

1 Take 20 μL of the homogenate and spread it evenly on the surface of a solid MRS medium plate.

2 Incubate the plate in a 37 °C incubator for 48 h.

**Figure 4:**
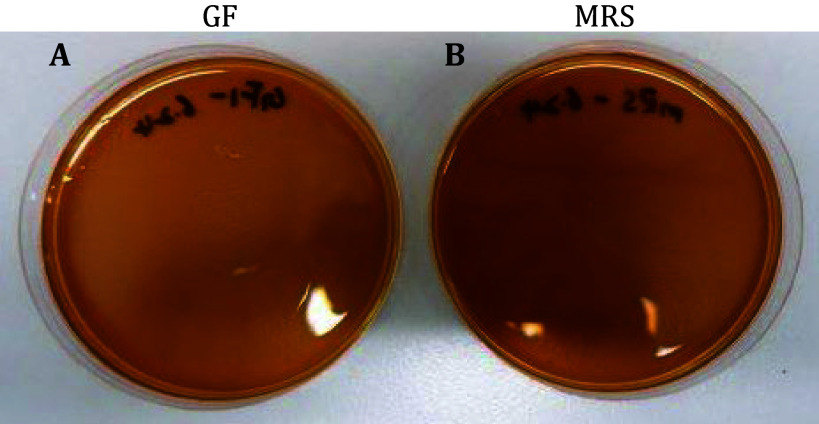
Verification of germ-free fruit fly. **A** Results of GF fly homogenate coated plate. **B** Results of MRS coated plate

3 After 48 h, remove the plate from the incubator and observe for colony growth. A successful model shows no colonies (Fig. 4).

## LIMITATION

The utilization of bio-reaction tubes equipped with a 0.22 μm filter membrane cap has significantly streamlined the traditional sterile rearing process for GF flies and enhanced the efficiency of model construction. However, despite its notable benefits, several potential limitations should be considered. Firstly, while these tubes help minimize microbial contamination, they do not eliminate the risk, especially during tube replacement. Secondly, this innovative method may increase experimental costs, as the bio-reaction tubes with a 0.22 μm filter membrane cap are typically more expensive than conventional sterile centrifuge tubes. Additionally, for experiments requiring higher levels of sterility, further validation and assessment of this approach's effectiveness and safety may be necessary. Therefore, while this method offers substantial advancements, researchers must comprehensively evaluate its suitability and constraints within their specific research contexts to ensure the precision and reliability of their experimental outcomes.

## CONCLUSION

Traditional methods for cultivating germ-free (GF) fruit fly models involve using sterile centrifuge tubes with sterile food, requiring frequent loosening of caps to ensure adequate air exchange. However, this manual intervention introduces variability and increases the risk of microbial contamination, resulting in a success rate of 70% to 80% for GF flies. Here, we present a streamlined protocol for the stable cultivation of GF flies under laboratory conditions. Our innovation utilizes bio-reaction tubes with 0.22 μm filter membrane caps to feed and maintain GF flies. This protocol emphasizes stringent aseptic techniques, performing all procedures on ultra-clean workbenches and using autoclaved reagents and consumables. Implementing this method markedly enhances the stability and reproducibility of GF fly cultures, consistently achieving a 100% success rate in GF fly preparation.

## Conflict of interest

An-Qi Li, Sha-Sha Li, Hui-Lin Li, Lan-Lan Zhong, Guo-Bao Tian, Xin-Yuan Zhao and Qiao-Ping Wang declare that they have no conflict of interest.
